# Drying Regimes on Regenerated Cellulose Films Characteristics and Properties

**DOI:** 10.3390/membranes12050445

**Published:** 2022-04-20

**Authors:** Nur Ain Ibrahim, Kushairi Mohd Salleh, Ahmad Fudholi, Sarani Zakaria

**Affiliations:** 1 Bioresources and Biorefinery Laboratory, Faculty of Science and Technology, Universiti Kebangsaan Malaysia, Bangi 43600, Selangor, Malaysia; aienib1607@gmail.com; 2 Solar Energy Research Institute, Universiti Kebangsaan Malaysia, Bangi 43600, Selangor, Malaysia; a.fudholi@ukm.edu.my

**Keywords:** activation energy, drying model, effective diffusion

## Abstract

Abundant water content and its interaction with cellulose macromolecules through hydrogen bonding engenders a complex drying process, the circumstances of which have not yet been unveiled. For instance, excessive drying on regenerated cellulose membranes (RCM) causes cracking and severe shrinking, affecting the produced regenerated cellulose film (RCF). Thus, mathematical models in estimating the drying kinetics and required energy to dry RCM are necessary. This study evaluated two drying techniques of oven drying and infrared (IR) drying on RCM at different temperatures of 50–80 °C. Five mathematical models were used, namely Newton, Page, Handreson–Pabis, logarithmic, and Wang–Singh, to adjust the obtained experimental data and were statistically validated using ANOVA to review their effect on the quality of the produced RCF. A logarithmic model and a Wang–Singh model were the best models for oven drying and IR drying of RCM, respectively. It was found that the physical property of the RCF was similar to all drying types. Meanwhile, for mechanical properties, the high temperature of oven drying affected the tensile properties of RCF compared with IR drying. This study is beneficial by approximating the drying kinetics of RCM and defining appropriate drying conditions, which controls the quality of its predictive physical and mechanical properties.

## 1. Introduction

Cellulose is a natural polymer mainly found in plants that exemplifies distinctive properties, such as non-toxic, hydrophilic, and outstanding mechanical and environmentally friendly properties [1,2,3]. Cellulose is broadly used as a renewable source in various applications as an alternative to petroleum-based materials such as bioplastic, biofilm, and biomembrane. However, its extensive applications do not use native cellulose but rather its modified structure. Even though cellulose is hydrophilic, the strong intra- and intermolecular hydrogen bonding of cellulose macromolecules has made them insoluble in water and a common solvent [3,4]. Thus, one of the most effective routes to dissolve cellulose is via aqueous alkaline/urea [3]. The dissolved cellulose can then be regenerated into various products depending on the regeneration process, such as hydrogel, beads, cellulose membrane, etc. [5,6]. The dissolving process of cellulose is often accomplished in aqueous media, where the produced regenerated cellulose products are bounteous with the water molecules [3,5,7]. Generally, the regenerated cellulose product develops a different molecular arrangement than its precursor, which is differentiated by its lattice structures, later identified as cellulose II, III, IV, and V [3,7]. Among the regenerated cellulose products, regenerated cellulose membrane (RCM) is our primary interest. It is physically approximately 0.018–0.020 mm thick, containing more than 90% water with a moist surface, being opaque, non-foldable, and easily torn when stretched [8]. The RCM is then identified as regenerated cellulose film (RCF) via the drying process. This film is approximately 0.0018–0.0020 mm thick, ≤10% water, transparent, foldable, and mechanically more robust than the RCM [8]. A film made from cellulose has been extensively developed for electronic and packaging applications due to its low thermal expansion coefficient, small pore size, transparency, and considerable tensile strength, and can be biodegraded to CO_2_ and H_2_O after being discarded [9,10]. Nevertheless, the consequential effect of different drying techniques was seldom reported.

Drying is a typical technique to improve long-term storage, transportation costs, and manageability. In the case of RCMs, drying is required as a key step to meet its targeted applications for thin material technology [3,6]. However, the drying of RCMs is a complex task because it often results in uncontrollable side effects, which meddles with their mechanical performance and physical structure [11,12]. Therefore, mitigating the drawbacks caused by drying processes must prevail. Although air drying is the most common method to dry RCMs, it takes a long time to dry the RCM [13]. Therefore, further evaluations and understanding of the drying process of RCMs are crucial to uplift the potential of natural products, especially cellulose, in high-end applications.

A previous study showed that air-dried RCF could be prepared using kenaf core cellulose [14]. However, the effect of the drying process has always been neglected and failed to be reported. Thus, the mathematical models’ approach to approximating kinetics and drying energy can help define the appropriate drying conditions, particularly of the RCM. Consequently, an efficient drying system and proper equipment can be manufactured. Thus far, several researchers have used mathematical drying models for food and agricultural products [15,16,17,18,19,20,21]. Unfortunately, investigations on the drying kinetics of cellulose-based RCMs have not been addressed in depth, although the bio-based product has recently become a primary concern worldwide.

The objective of this work is (a) to study the drying kinetics; (b) to estimate two types of mathematical modeling, namely semi-theoretical (i.e., Newton, Page, and Henderson–Pabis, logarithmic) and empirical models (i.e., Wang–Singh), to fit the experimental drying data; and (c) to explore the effect of drying types and conditions, including effective diffusion, activation energy, and the physical and mechanical properties. The outlined objectives aim to reduce the RCM drying time without degrading the physical and mechanical properties of the produced RCF. This work will be beneficial, particularly for dissolution–regeneration technology of cellulose-based products in an unlimited array of applications. 

## 2. Materials and Methods

### 2.1. Materials

Kenaf core soda pulp (25% alkaline active) was supplied by the Forest Research Institute Malaysia (FRIM) (Kuala Lumpur, Selangor, Malaysia). Sodium hydroxide (NaOH), urea, and sulfuric acid (H_2_SO_4_) of analytical grade were acquired from R&M.

### 2.2. Fabrication of Regenerated Cellulose Membrane

The RCM was prepared based on the previous study [14]. After the cellulose was added to the aqueous NaOH/urea solvent at −13 °C, the cellulose solution was dynamically stirred to achieve a homogeneous solution. The cellulose solution was cast and coagulated in the coagulation bath of 5% H_2_SO_4_ to initiate the formation of RCM. The RCM was then neutralized before proceeding with the drying process.

### 2.3. Regenerated Cellulose Membrane Drying Procedures

The RCM was dried under two drying methods, oven drying and IR drying, performed at similar temperatures of 50, 60, 70, and 80 °C. Both dryers were preheated for approximately half an hour to achieve a steady state before the RCM was dried. The RCM was spread on a tray, with 5 × 5 cm dimensions, and placed inside the dryer. The dried RCM is known as RCF. Oven drying the RCF was accomplished with forced circulation. Meanwhile, IR drying the RCF was conducted in the drying chamber, installed with a 3000 W electric heater inside. 

The weight changes in the RCM samples were recorded for every minute interval until they reached the equilibrium weight. All measurements were carried out in triplicate. The moisture content (MC) percentage was calculated at every minute interval. The equation for the MC of RCF on a dry basis was expressed as:

(1) $$\mathrm{MC}\;\left(\%\right)=\frac{\left(\mathrm{weight}\;\mathrm{of}\;\mathrm{wet}\;\mathrm{RCM}\;-\;\mathrm{weight}\;\mathrm{of}\;\mathrm{dry}\;\mathrm{RCM}\right)}{\mathrm{weight}\;\mathrm{of}\;\mathrm{dry}\;\mathrm{RCM}}\times 100$$

### 2.4. Mathematical Modeling of Drying Curve 

As shown in Table 1, five mathematical models were adopted to describe the drying curve equation of the RCF, and the best model was determined. Two types of mathematical models, namely semi-theoretical and empirical models, were used. Semi-theoretical models (i.e., Lewis, Page, Henderson–Pabis, and logarithmic) were attributed to the approximately estimated theoretical equation and are only valid at a given temperature, airflow velocity, relative humidity, and range of MC percentage during the experiment. On the other hand, the empirical model (i.e., Wang–Singh) depended on the experimental data. These models revealed the drying curve of various drying methods of RCF, but the fundamentals and drying methods had no physical meaning. The external resistance to the moisture movement of RCM was the key factor for the two types of models (semi-theoretical and empirical models). Thus, both types of models are habitually used to model the drying curve.

The moisture ratio (MR) of the RCM during drying is calculated using the equation:

(2) $$\mathrm{MR}=\frac{M}{M_{i}}$$

where M (g water/g dry solid) is the moisture content at any time, and M_i_ (g water/g solid) is the initial MC.

The employed mathematical models on the experimental data were evaluated by using a coefficient of determination (R^2^), reduced chi-square (X^2^), and root mean square error (RMSE). High R^2^ values, and low X^2^ and RMSE values, represent a better fit. R^2^, X^2^, and RMSE equations are expressed as follows:

(3) $$R^{2}=\frac{\sum_{i=1}^{N}{\left({\mathrm{MR}}_{\exp.i}{-\mathrm{MR}}_{\mathrm{pre}.i}\right)}^{2}}{\sqrt{\left[\sum_{i=1}^{N}{\left({\mathrm{MR}}_{\exp.i}{-\mathrm{MR}}_{\mathrm{pre}.i}\right)}^{2}\right]*\left[\sum_{i=1}^{N}{\left({\mathrm{MR}}_{\exp.i}{-\mathrm{MR}}_{\mathrm{pre}.i}\right)}^{2}\right]}}$$

(4) $$X^{2}=\frac{\sum_{i=1}^{N}{\left({\mathrm{MR}}_{\exp.i}{-\mathrm{MR}}_{\mathrm{pre}.i}\right)}^{2}}{N-z}$$

(5) $$\;\mathrm{RMSE}={\left[\frac{1}{N}\overset{N}{\underset{i=1}{\sum }}{\left({\mathrm{MR}}_{\mathrm{pre}.i}{-\mathrm{MR}}_{\exp.i}\right)}^{2}\right]}^{\frac{1}{2}}$$

where ${\mathrm{MR}}_{\exp.i}$ is the *i*th experimental MR, ${\mathrm{MR}}_{\mathrm{pre}.i}$ is the *i*th predicted MR, and N is the number of observation constants.

### 2.5. Determination of Effective Diffusion Coefficients

Fick’s diffusion equation can illustrate the falling rate period of dried RCF products [27]. Fick’s diffusion equation was developed by considering the moisture released by diffusion, constant temperature, diffusion coefficient, and long drying times by ignoring the shrinkage of the samples [28]. Therefore, this equation can be applied to various shapes of products. In this study, Fick’s diffusion equation for rectangular geometry was used to determine the MR, as shown in Equation (6):

(6) $$\mathrm{MR}=\frac{8}{\pi^{2}}\overset{\infty }{\underset{n=0}{\sum }}\frac{1}{\left(2n+1\right)}\exp\left(-\frac{\left(2n+1\right)\pi^{2}}{L^{2}}D_{\mathrm{eff}}t\right)$$

where D_eff_ is the effective diffusivity (m^2^/s)v and L is the thickness of samples (m); n is a positive integer.

Equation (7) is simplified from Equation (6) for a long drying time:

(7) $$\mathrm{MR}=\frac{8}{\pi^{2}}\exp\left(-\frac{\pi^{2}D_{\mathrm{eff}}t}{L^{2}}\right)$$

For the slope of graph ln MR versus drying time, t gives the drying constant (k_0_), as shown in Equation (8), and the diffusion coefficient (D_eff_) is determined.

(8) $$k_{0}=\frac{\pi^{2}D_{\mathrm{eff}}}{L^{2}}$$

Activation energy (E_a_) in kJ/mol is calculated using Equation (9).

(9) $$D_{\mathrm{eff}}{=D}_{o}\exp\left(\frac{{-E}_{a}}{\mathrm{RT}}\right)$$

where D_0_ is the pre-exponential factor, R is the universal gas constant (8.314 J/K/mol), and T is the temperature in kelvin (K).

### 2.6. Determination of Film Thickness and Density

The thickness of dried RCF was measured using a precision digital micrometer to the nearest 0.0001 (±5%) at five different points on the sample, four at the edge regions and one in the central area. In addition, the average thickness value of one RCF sample was calculated. The density of the dried RCF was determined based on its dry weight over the volume of the samples.

### 2.7. Swelling and Pore Volume

The swelling percentage of the dried RCF was measured through a re-wetting test at room temperature. The swelling percentage (Q) is calculated by using Equation (10) [14]:

(10) $$\;Q\left(\%\right)=\frac{W_{\mathrm{wet}}{-W}_{\mathrm{dry}}}{W_{\mathrm{dry}}}\times 100$$

where Q is the percentage of film swelling, W_wet_ is the weight of the swollen film, and W_dry_ is the weight of the dried film.

The pore volume (V_p_) of the dried RCF was measured using a room temperature re-wetting test. The V_p_ of the dried RCF is calculated using Equation (11) [29]:

(11) $$V_{p}=\frac{W_{\mathrm{wet}}{-W}_{\mathrm{dry}}}{{\rho \times \;W}_{\mathrm{dry}}}$$

where W_wet_ is the weight of the swollen film, W_dry_ is the weight of the dried film, and the density of water (ρ) is 0.998 g/cm^3^ at 30 °C [27].

### 2.8. Tensile Properties

The tensile strength and elongation at the break of the dried RCM were measured using a GOTECH, model AI-3000 at 10 mm min^−1^. The samples were cut into a size of 8 × 1 cm. Five replicates for each sample were completed. The tensile properties were calculated using the following equations:

(12) $$\mathrm{Tensile}\;\mathrm{strengh}=\frac{F_{\max}}{t\;\times \;w}$$

where F_max_ is the load at failure (force at which films break), t is the initial film thickness, and w is the initial film width.

(13) $$\mathrm{Percent}\;\mathrm{elongation}\;\mathrm{at}\;\mathrm{break}=\frac{I_{f}{-\;I}_{0}}{I_{0}}\;\times \;100$$

where I_f_ is the final length of the film at failure and I_0_ is the initial length of the film between grips.

### 2.9. Statistical Analysis

The results are presented as mean values. The data were validated using ANOVA, and significant differences were compared using Tukey’s method (confidence level *p* < 0.05).

## 3. Results

### 3.1. Drying Characteristic of Cellulose Membrane

**Figure 1:**
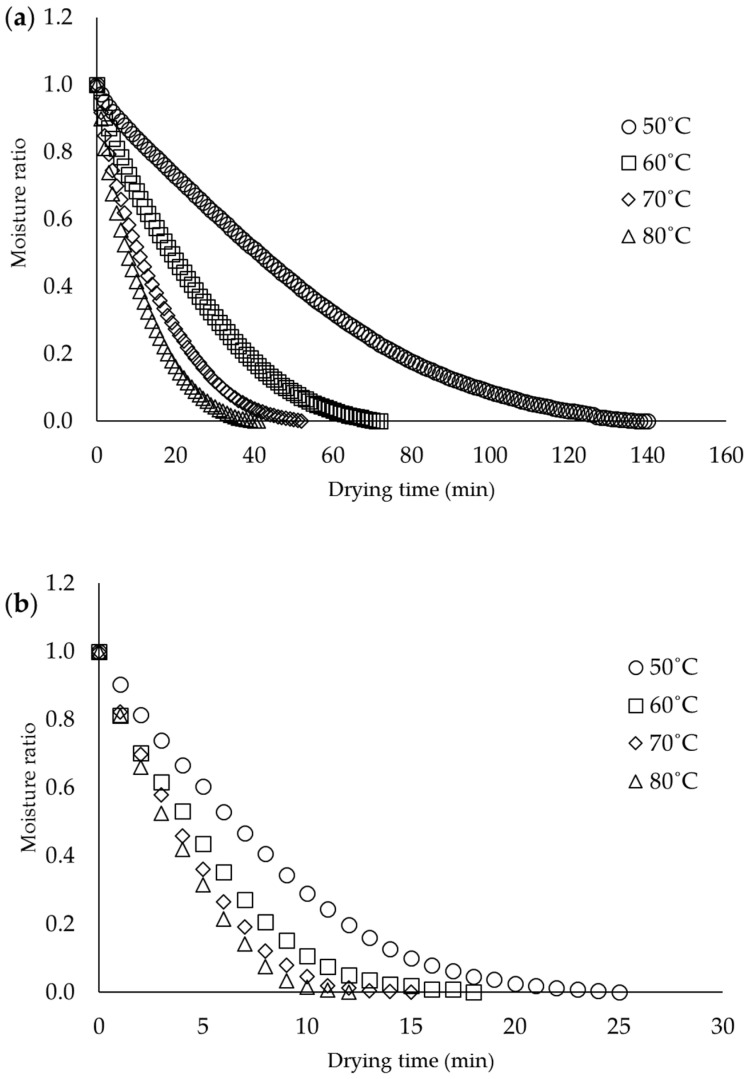
Variation in MR with drying time at various (**a**) oven drying and (**b**) IR drying temperatures.

The RCM with an initial MC value of ~92% was dried using two different drying methods: oven drying and IR drying. The primary purpose for both dryings is to reach an equilibrium MC on the RCF. To obtain accurate drying times of the RCF to reach an equilibrium weight is by predetermining the initial MC of the RCF. By doing this, cracking and warping on the RCF can be avoided. A graph of the MR versus drying time curve, for oven drying and IR drying, of the RCM under various temperatures is shown in Figure 1a,b, respectively. As noted in Figure 1a,b, the MR of all samples, based on different drying temperatures, decreased over drying time. An increment in drying temperature accelerates the drying time for both drying types. This has proven that the drying power increases by increasing the drying temperature [30]. Rapid moisture evaporation on the RCM at higher temperatures is due to the increment in water molecule energy and large differences in the partial vapor pressure between drying air and moisture vapor pressure in the RCM [31]. The drying rate of the RCM from 92% MC to its equilibrium depended on water state changes in the sample. For instance, at the beginning of the drying process, evaporation involving the diffusion of additional water, known as free or bulk water molecules, imbibe in the RCM structure. Due to their “free” state, they easily diffused out from the RCM structure upon the drying process, which subsequently increased the drying rate. When the free water molecule is fully evaporated, the remaining primary and secondary bound water requires higher drying energy to be evaporated [5]. This event instigated the RCM to have a slower drying rate that entails a longer drying time. This is because the samples are dominated by bound water molecules, not free water molecules. Unlike free water, bound water molecules require a higher energy to escape and diffuse out from the RCM matrix, making the drying time of the RCM longer [32]. As seen in Figure 1a,b, both drying processes mimic a similar trend, where higher temperatures showed a higher drying rate with a steeper slope. 

**Figure 2:**
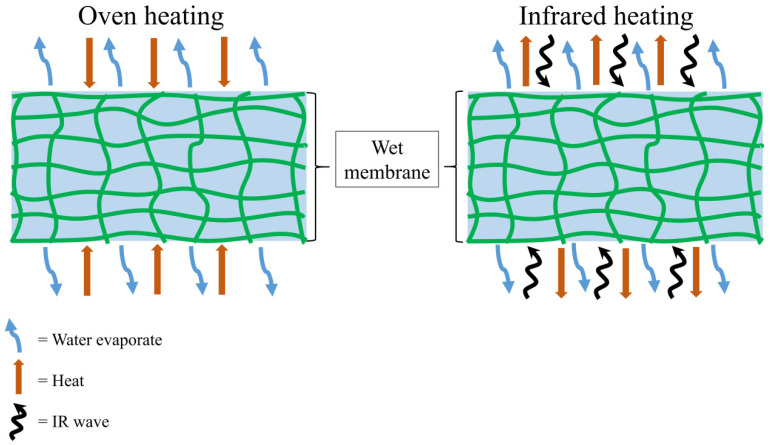
Schematic representation of the heating mechanisms of oven drying and IR drying on RCM.

The required times for the MC removal of the RCF to reach equilibrium weight, at drying temperatures of 50, 60, 70, and 80 °C, for oven drying were 140, 72, 52, and 38 min, and IR drying were 28, 26, 17, and 11 min, respectively. From the results, the IR drying took less time to dry than oven drying. This is because IR radiation energy, through the radiant process, is transmitted from the source to the surface of the RCM without heating the surrounding air. IR radiation imposes on the exposed RCM, penetrating and converting it to a sensible heat [33]. IR energy absorption by the RCM depends largely on the MC, which affects the drying kinetics. Due to this, IR drying is widely used to increase productivity and reduce operating costs as it requires a shorter drying time and does not affect the quality of the end product [34]. Meanwhile, oven drying is a convective drying process in which convection occurs when the heat is transferred from the hot air to the samples, followed by the evaporation process. The evaporation kinetics of water in conventional drying is influenced by the mass transfer of the materials and heat resistance. This type of drying takes a longer time and may cause damage to the heated materials [35]. The schematic representation of the heating mechanisms of oven drying and IR drying on the RCM for the heat and mass transfer process is pictorially summarized in Figure 2.

### 3.2. Evaluation of the Models

The experimental data of the drying process were fitted into the mathematical models as listed in Table 1. R^2^, X^2^, and RMSE were used to evaluate the drying models to characterize the experimental drying curves. Table 2 shows the statistical analysis of the drying models for the oven drying and IR drying of RCF. Among the five models, the logarithmic model best fits the oven drying data, due to its higher R^2^ value and lower X^2^ and RMSE values than the other models. Therefore, the logarithmic drying constant becomes:

a = 0.3633ln (T) − 1.6256
b = −0.438ln (T) + 2.9219
k = 0.0023T − 0.101

The Wang–Singh model is fitted with an IR drying curve, having the highest R^2^ values and low X^2^ and RMSE values. Therefore, the Wang–Singh drying constant becomes:

a = 0.0002T − 0.008
b = −0.19ln (T) + 0.6522

**Figure 3:**
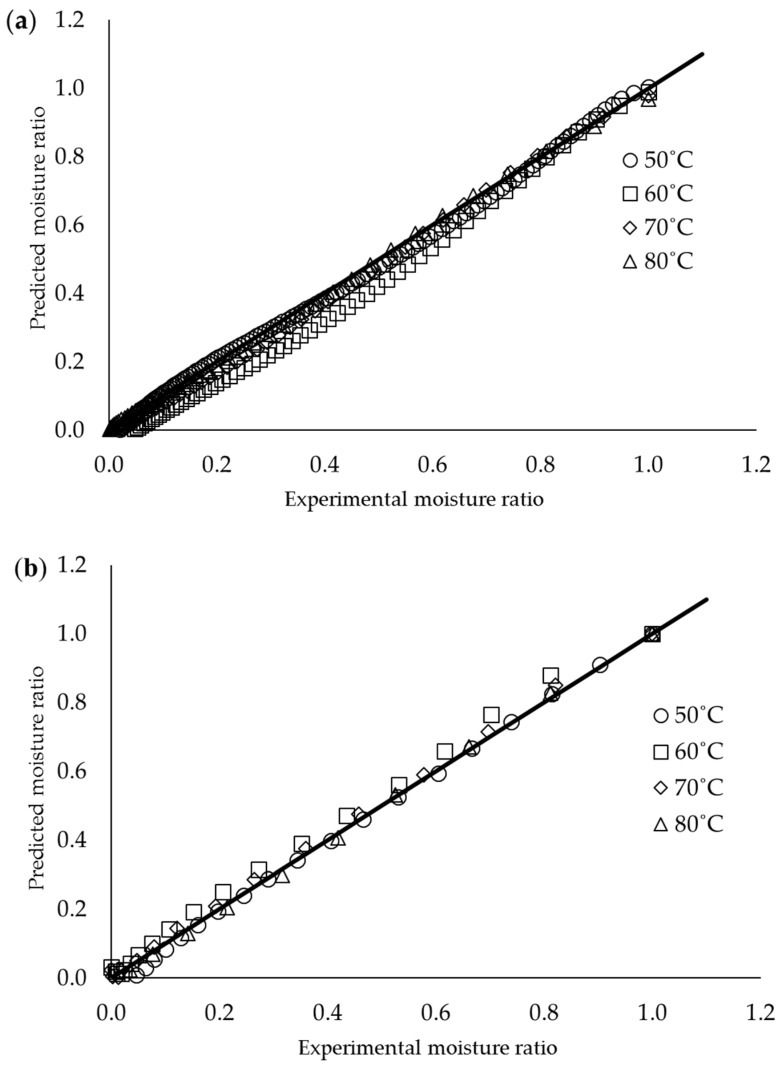
Predicted MR versus experimental MR under different drying temperatures: (**a**) oven drying logarithmic model, and (**b**) IR drying Wang–Singh model.

The predicted and experimental MR plots are presented in Figure 3 to illustrate the performance of the logarithmic model for oven drying and the Wang–Singh model for IR drying. The logarithmic model for oven drying showed a straight line with an R^2^ of 0.9991, while the Wang–Singh model for IR drying showed a straight line with an R^2^ of 0.9882. These findings indicate that these models are highly suitable to predict the drying characteristics of the RCF in the conducted experimental range of this study. These two models are widely used on high initial MC samples of thin-layer drying studies. Furthermore, it was proven from previous studies that these models are fitted in predicting the drying of corn ears [36], apricot [37], hull-less seed pumpkin [38], and sweet sorghum [18].

### 3.3. Effective Diffusion and Activation Energy

The average effective moisture diffusivity (D_eff_) is provided in Equation (8). The determined average values of D_eff_ for oven drying and IR drying are listed in Table 3. Hypothetically, the temperature will influence the D_eff_ values. From Table 3, it can be seen that the D_eff_ values were increased when the temperature was high. This is due to the increment in heating energy ensued with the increment in the vibration frequency of water molecules, resulting in the high fluid diffusion of the RCF [36]. The results show that the D_eff_ of IR drying was higher than oven drying. This condition is due to the IR waves penetrating the internal part of the wet membrane and converting to thermal energy, which provides a rapid and efficient heating mechanism to the RCM [27]. Unlike the oven drying process, heating involves heated air as the medium that produces double diffusions externally and internally after the RCM absorbs the heat. Thus, double diffusions caused the decrement in D_eff_ values.

**Figure 4:**
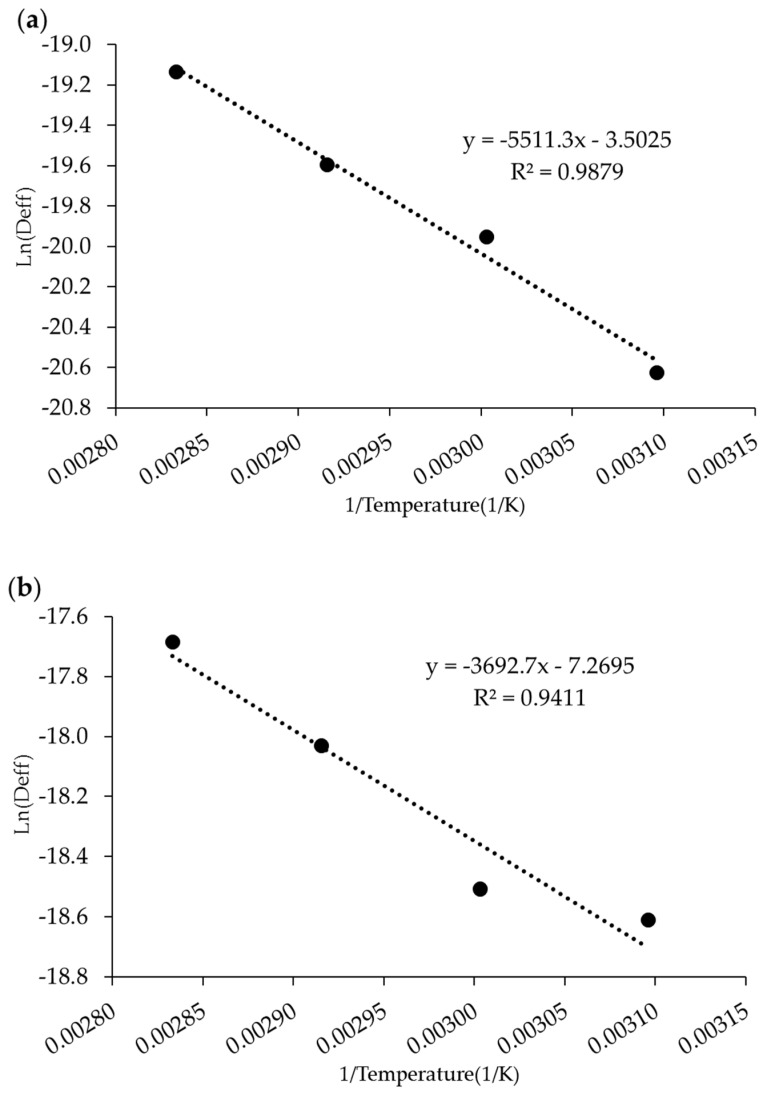
Relationship plot between ln(D_eff_) versus reciprocal drying temperature for (**a**) oven drying and (**b**) IR drying.

Figure 4 shows the ln(D_eff_) graph versus reciprocal temperature (expressed in units of K). This graph presented a linear relationship with a high correlation coefficient (R^2^ = 0.9879 and 0.9441). Using Equation (9), the activation energy value for the oven-dried RCF was 45.82 kJ/mol, which is higher than that of IR-dried RCF (30.7 kJ/mol). In the drying process, the lower the activation energy, the higher the diffusivity of the product’s water [39]. Thus, in our case, the RCF obtained from IR drying has a higher water diffusivity than that of the RCF dried with an oven drying process in line with the drying rate and drying mechanism as stated in Section 3.1.

### 3.4. Characterization of Dried RCF

**Figure 5:**
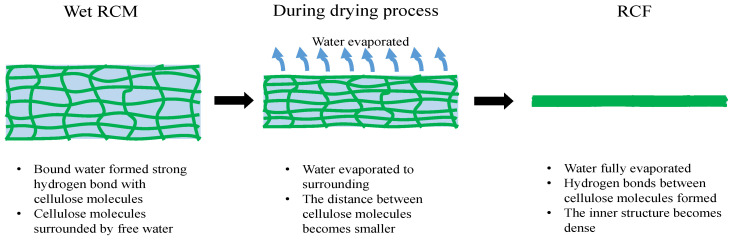
Schematic of the drying process and its effect on RCF molecular structure arrangement.

The physical properties of the dried RCF are shown in Table 4. The initial value of the thickness is ~0.25 mm, and density is ~1.3 gcm^−3^. The air-dried RCF was used as the control sample. The density, thickness, pore volume, and swelling percentages values for all samples were statistically similar to the values of the control sample (*p* > 0.05). During the RCF drying process, the interconnection interactions of cellulose macromolecules initially collapsed and formed strong hydrogen bonds between hydroxyl groups [12]. Therefore, the dried RCF became dense. Hence, the physical properties of the RCF are similar between air drying, oven drying, and IR drying. It was found that the oven drying and IR drying methods decrease the drying time without affecting the physical properties of the RCF. Figure 5 shows the schematic of the drying effect on RCF.

### 3.5. Tensile Strength

The tensile strength and elongation at break are typically evaluated to describe the mechanical properties of an RCF [40]. It is a known fact that the mechanical properties of RCFs are related to their internal structure. The effects of the drying technique and drying temperatures on the mechanical properties of cellulose films were explored and are presented in Table 5. Control samples have a tensile strength value of 59.4 MPa and elongation at a break of 5.561%. The results show that a significant difference (*p* < 0.05) was observed in the tensile strength of the RCF at 70 and 80 °C for oven drying. The decrement in the tensile strength value at 70 and 80 °C might be due to the higher drying temperature and time taken in the oven, which led to the higher mobility of water molecules. Hence, few regularities in the formation of intra- and intermolecular hydrogen bonds occurred on the RCF. During the IR drying process, the decrement in the water content occurred in a shorter time because the effective energy transfer quickened the water molecules’ escape from the RCM matrix structure. Continuous evaporation on the RCM makes the shrinking process inevitable. Upon this, the effective surface area and pore volume of the RCM are also decreased. According to the different drying processes and the length of time the product is exposed to heat, these related events led to different behavioral changes in the RCM. Nevertheless, for the elongation, based on different drying conditions, there was no significant difference (*p* > 0.05) observed on the RCF.

## 4. Conclusions

This study revealed the effect of drying conditions on the drying behavior of RCFs through oven drying and IR drying. The drying rate remarkably increased with temperature for both types of drying. The logarithmic model was the best drying model for predicting the drying behavior of oven-dried RCF, and the Wang–Singh model was suitable for predicting the IR-dried RCF. The R^2^ values were approaching one for both drying methods, X^2^ and RMSE values were approaching zero. IR-dried RCF had a high water diffusivity and low activation energy compared with oven-dried RCF. Both drying types embodied similar physical properties to air-dried RCF. However, a discrepancy was observed in the mechanical properties of oven-dried RCFs with an apparent decrement in the tensile value at 70 and 80 °C, while IR drying did not cause a similar impact. Therefore, it can be concluded that IR drying and oven drying below 60 °C is an effective method to reduce the drying time of RCFs and produces unaffected physical and tensile properties, portraying similar characteristics to air drying.

## Figures and Tables

**Table 1:** Mathematical models and equations employed for RCM drying.

No.	Model	Equation	References
1	Newton	MR = exp (−kt)	Lewis [22]
2	Page	MR = exp (−kt^n^)	Page [23]
3	Henderson–Pabis	MR = a.exp (−kt)	Henderson and Pabis [24]
4	Logarithmic	MR = a + bexp (−kt)	Degirmencioglu et al. [25]
5	Wang–Singh	MR = at^2^ + bt + 1	Wang and Singh [26]

where t = drying time, s; k = drying constant, s^−1^; a, b, n = model parameters, dimensionless.

**Table 2:** R^2^, X^2^, and RMSE values of the drying curve of oven-dried and IR-dried RCM.

Drying Type	Model	Temperature (°C)	R^2^	X^2^	RMSE
Oven drying	Newton	50	0.973764	0.320098	0.047647
		60	0.984203	0.093485	0.035786
		70	0.994375	0.022085	0.020413
		80	0.995616	0.013764	0.018103
	Page	50	0.994375	0.068628	0.022062
		60	0.992743	0.042943	0.024254
		70	0.995135	0.019101	0.018984
		80	0.995990	0.012590	0.017313
	Henderson–Pabis	50	0.979333	0.252150	0.042288
		60	0.985545	0.085543	0.034232
		70	0.994379	0.022070	0.020406
		80	0.995616	0.013764	0.018103
	Logarithmic	50	0.997589	0.029413	0.014443
		60	0.998712	0.007624	0.010220
		70	0.999195	0.003161	0.007723
		80	0.998957	0.003276	0.008832
	Wang–Singh	50	0.999366	0.007740	0.007409
		60	0.995262	0.028038	0.019598
		70	0.977392	0.088762	0.040924
		80	0.971319	0.090051	0.046304
IR drying	Newton	50	0.978146	0.053920	0.045540
		60	0.974252	0.048225	0.050380
		70	0.979273	0.033343	0.045650
		80	0.977783	0.029974	0.048018
	Page	50	0.996858	0.007751	0.017266
		60	0.995240	0.008915	0.021661
		70	0.995642	0.007011	0.020933
		80	0.994323	0.007659	0.024273
	Henderson–Pabis	50	0.963677	0.041575	0.039988
		60	0.978961	0.039406	0.045541
		70	0.982395	0.028322	0.042073
		80	0.980801	0.025902	0.044637
	Logarithmic	50	0.995670	0.010684	0.020271
		60	0.996523	0.006503	0.018501
		70	0.994941	0.008139	0.022554
		80	0.996735	0.004405	0.018408
	Wang–Singh	50	0.999068	0.002299	0.009404
		60	0.999438	0.001053	0.007446
		70	0.998610	0.002236	0.011821
		80	0.999379	0.000838	0.008031

**Table 3:** Values of effective diffusivity obtained for RCM drying at different temperatures.

Sample	Temperature (°C)	D_eff_ (m^2^/s)	Ea (kJ/mol)
Oven drying	50	1.10567 × 10^−9^	45.82
	60	2.16122 × 10^−9^	
	70	3.09148 × 10^−9^	
	80	4.89876 × 10^−9^	
IR drying	50	8.28781 × 10^−9^	30.70
	60	9.18049 × 10^−9^	
	70	1.47903 × 10^−8^	
	80	2.09231 × 10^−8^	

**Table 4:** Thickness, density, pore volume, and swelling percentage for dried RCF.

Sample	Temperature (°C)	Thickness (mm)	Density (g/cm^3^)	V_p_ (cm^3^/g^1^)	Swelling Percentage (%)
Oven drying	50	0.020 ^a^	1.277 ^a^	1.106 ^a^	110.418 ^a^
	60	0.020 ^a^	1.237 ^a^	1.163 ^a^	116.164 ^a^
	70	0.020 ^a^	1.291 ^a^	1.106 ^a^	110.423 ^a^
	80	0.020 ^a^	1.303 ^a^	1.162 ^a^	115.966 ^a^
IR drying	50	0.020 ^a^	1.243 ^a^	1.157 ^a^	115.458 ^a^
	60	0.019 ^a^	1.300 ^a^	1.090 ^a^	108.763 ^a^
	70	0.018 ^a^	1.245 ^a^	1.125 ^a^	112.284 ^a^
	80	0.020 ^a^	1.283 ^a^	1.153 ^a^	115.143 ^a^
Air drying	Room temperature	0.018 ^a^	1.267 ^a^	1.140 ^a^	113.805 ^a^

Data are expressed as in means values. Mean values in the same column with different superscript letters are significantly different (*p* < 0.05).

**Table 5:** Tensile strength and elongation for dried RCF.

Sample	Temperature (°C)	Tensile Strength (MPa)	Elongation (%)
Air drying	Room temperature	59.402 ^a^	5.561 ^a^
Oven drying	50	63.107 ^a^	5.196 ^a^
	60	66.009 ^a^	5.409 ^a^
	70	50.108 ^b^	5.316 ^a^
	80	45.414 ^b^	6.104 ^a^
IR drying	50	64.953 ^a^	5.221 ^a^
	60	62.453 ^a^	5.875 ^a^
	70	62.205 ^a^	5.552 ^a^
	80	63.703 ^a^	5.873 ^a^

Data are expressed in mean values. Mean values in the same column with different superscript letters are significantly different (*p* < 0.05).

## Data Availability

Not applicable.
